# The relationship between postnatal hypoglycemia and umbilical artery Doppler ultrasonography in neonates with intrauterine growth restriction: A longitudinal follow-up study

**DOI:** 10.18502/ijrm.v20i2.10505

**Published:** 2022-03-21

**Authors:** Razieh Sadat Tabatabaie, Naeime Dehghan, Mahdieh Mojibian, Mohamad Hosein Lookzadeh, Nasim Namiranian, Atiyeh Javaheri, Mojgan Hajisafari

**Affiliations:** ^1^Department of Obstetrics and Gynecology, Shahid Sadoughi University of Medical Sciences, Yazd, Iran.; ^2^Mother and Newborn Health Research Center, Shahid Sadoughi University of Medical Sciences, Yazd, Iran.; ^3^Community and Preventive Medicine, Yazd Diabetes Research Center, Shahid Sadoughi University of Medical Sciences, Yazd, Iran.

**Keywords:** Growth restriction, Hypoglycemia, Ultrasonography Doppler, Umbilical artery.

## Abstract

**Background:**

Intrauterine growth restriction (IUGR) refers to fetuses with an estimated ultrasonography weight below the 10% percentile. Hypoglycemia is a major concern in neonates with IUGR.

**Objective:**

To investigate the relationship between umbilical artery (UA) Doppler ultrasonography and neonate hypoglycemia and IUGR.

**Materials and Methods:**

This was a longitudinal follow-up study consisting of 114 neonates (gestational age of 28-40 wk) born with IUGR in the third trimester of pregnancy at Shahid Sadoughi Hospital, Yazd, Iran between May 2016 and October 2017. The neonates were assigned into three subgroups of normal UA Doppler, absent end-diastolic flow (EDF) in UA Doppler, and reverse EDF in UA Doppler. The blood glucose of the neonates was checked one, two, three, six, 12, 24 and 48 hr after birth, and the neonates were placed in the hypoglycemia or euglycemia groups according to guidelines.

**Results:**

Out of the 114 neonates included in the study, 75 (65.8%) had normal UA Doppler, 29 (25.4%) had absent EDF in UA Doppler, and 10 (8.8%) had reverse EDF in UA Doppler. There was a significant difference in the mean blood glucose in the first hr between the normal UA Doppler group and the reverse EDF in UA Doppler group (p < 0.01).

**Conclusion:**

Postnatal hypoglycemia in neonates with IUGR is associated with the result of UA Doppler ultrasonography during pregnancy.

## 1. Introduction 

Intrauterine growth restriction (IUGR) or fetal growth restriction is a condition in which the fetus (unborn baby) is unable to achieve their potential and expected growth based on their gestational age due to environmental or genetic factors (1, 2). The placenta plays a key role as a mediator in the relationship between the mother and fetus in embryo development. Therefore, fetal insufficiency for any reason can be the main mechanism of growth restriction. Fetal insufficiency leads to a negative effect on general blood nutrients and active oxygen exchange and reduces the synthesis of glucose, proteins, and lipids (3, 4). At the time of delivery, newborns with IUGR usually look like the gestational age of an unborn baby (5).

The most common complications of growth restriction include hypoglycemia, asphyxia, hypoxic-ischemic encephalopathy, gastrointestinal bleeding, polycythemia, maternal malformations, pulmonary bleeding, apnea, congenital heart diseases, and disseminated intravascular coagulation. Growth restriction is also associated with higher morbidity and mortality (6, 7). Therefore, research on IUGR is a global priority.

Umbilical artery (UA) Doppler indicates the resistance in blood perfusion at the placenta-fetus level (8). Placental and maternal conditions can lead to the closure of small muscular arteries in tertiary chorionic villi, and progressive reduction in the end-diastolic flow (EDF), absent EDF, or reverse EDF in the UA Doppler (9). Reverse EDF in the UA blood flow indicates a developed condition of the fetus and may indicate a closure of more than 70% of the arteries in the tertiary chorionic villi (10, 11). Absent or reverse EDF in the UA is commonly associated with severe IUGR or oligohydramnios (12, 13). Therefore, in cases of suspected fetal growth restriction, UA Doppler is a reasonable measure. Although there are few methods available for UA Doppler, the systolic to diastolic ratio and pulsatility index are commonly used to treat suspected cases of IUGR (14).

Hypoglycemia is a major concern in neonates with growth restriction. Hypoglycemia reduces the level of consciousness and can lead to seizures followed by neonatal hypoxia (15). Therefore, blood glucose should be monitored in neonates with growth restriction. Maternal or fetal conditions which cause the closure of fetal small arteries, increase the EDF in the UA so that it finally causes absence and reverse EDF of the UA (16).

This study was conducted to evaluate the relationship between hypoglycemia (as a confirmed complication) in neonates with IUGR in the third trimester of pregnancy and type of UA Doppler.

## 2. Materials and Methods

In this longitudinal follow-up study, 114 infants born with IUGR in the third trimester of pregnancy at Shahid Sadoughi Hospital in Yazd, Iran from May 2016 to October 2017 were evaluated. Doppler UA was performed for all women up to 24 hr after delivery. This study included three subgroups of: normal UA, absent EDF in UA Doppler, and reverse EDF in UA Doppler. Neonates with risk factors for neonatal hypoglycemia were excluded from the study, including those with mothers with diabetes, severe neonatal hepatic or metabolic diseases, asphyxia during birth, meconium aspiration syndrome, or hypothermia.

The blood glucose of the neonates was checked during the first hr of birth using a glucometer (Zyklus Med TD-4267, Taiwan). Hypoglycemia was diagnosed if the blood glucose was < 30 mg/dl in the first hr. Blood glucose was then measured one, two and three hr after birth. According to guidelines (American Academy of Pediatrics 2011), the neonates were divided into the hypoglycemia or euglycemia groups (36). All ultrasounds were performed by one person (a perinatologist) using the same device (Mindray DC-8 3.5 MHZ probe, China). Normal UA, EDF, reverse EDF, and the amniotic fluid index (AFI) were evaluated.

It is worth noting that blood sugar was checked in all neonates in the first hr after birth, but blood sampling at the second and third hr was only done for neonates with abnormal blood sugar levels. For neonates with normal blood sugar in the first hr, sampling in the following hr was not performed.

### Ethical considerations

This study was approved by the Ethical Committee of Shahid Sadoughi University of Medical Sciences, Yazd, Iran (Code: IR.SSU.MEDICINE.REC.1396.61). Written informed consent was obtained from all of the parents. Blood sampling was done by a qualified nurse.

### Statistical analysis 

The data were analyzed using the Statistical Package for the Social Sciences (IBM SPSS Statistics for Windows, version 18, Armonk, NY, USA: IBM Corp). Data were expressed as mean ± standard error of the mean (SEM). P-values < 0.05 were considered statistically significant. Independent *t* tests and analysis of variance (ANOVA) were done to compare the mean and variance of variables. The normal distribution of the main variables was checked by the Shapiro Wilk test.

## 3. Results 

From a total of 138 neonates who were born in the third trimester of pregnancy and were diagnosed with IUGR using UA Doppler ultrasonography, 17 cases had mothers with maternal diabetes, two had neonatal metabolic disease, and five had meconium aspiration; these neonates were excluded from the study. Therefore, 114 neonates were evaluated. The mean age of the mothers and the gestational age based on the first day of last menstruation were 34.0 ± 3.2 yr and 28.3 ± 5.5 wk, respectively. The descriptive results of the studied variables are summarized in table I.

Based on UA Doppler ultrasonography, 29 and 10 neonates had absent EDF and reverse EDF, respectively. The results showed that there was a significant difference between the three Doppler groups in terms of mean blood glucose (p < 0.01). The frequency distribution based on UA Doppler is presented in table II.

There was a significant difference between the mean blood glucose in the first hr between the normal UA Doppler group and the reverse EDF in UA Doppler group (p < 0.01). No significant difference was seen between the normal UA Doppler and absent UA Doppler groups, or between the reverse UA Doppler and absent UA Doppler groups (p = 0.07 and p = 0.17, respectively, Tables II, III).

There was a significant difference in the first-hr blood glucose levels of neonates depending on the rate of AFI (borderline amniotic fluid [ < 5 cm] vs. normal [ > 8 cm]) (Table IV). There was a significant difference in the rate of AFI across the three groups of Doppler ultrasonography, and the difference was seen between the normal and reverse UA Doppler groups (p = 0.01, Table V).

There was no significant difference in the weight estimated by ultrasonography (EFW) between the three groups based on first-hr mean glucose level (p = 0.11, Table VI). There was no significant difference between the three groups of Doppler ultrasonography (p = 0.05).

**Table 1:** Descriptive results of the studied variables


**Variable**		**n**	**Min-max**	**Mean ± SD**
**Mother's age (yr)**		114	17-41	28.6 ± 5.5
**Gestational age (wk)**		114	28-40	34.0 ± 3.2
**Blood glucose**				
	**One hr after birth**	114	15-168	64.5 ± 31.3
	**Two hr after birth**	84	28-182	68.1 ± 28.3
	**Three hr after birth**	82	25-160	75.7 ± 23.8
Min: Minimum, Max: Maximum, SD: Standard deviation				

**Table 2:** Comparison of maternal age, gestational age, and mean blood glucose after birth based on umbilical artery Doppler ultrasonography


	**NUA**	**EDF**	**REDF**	p-value*
**Mother's age (yr)**	28.36 ± 4.20	28.48 ± 4.30	28.96 ± 5.80	0.96
**Gestational age (wk)**	34.5 ± 2.56	32.9 ± 3.14	35.5 ± 4.21	0.86
**Blood glucose after birth**	71.56 ± 3.50	56.34 ± 5.60	35.80 ± 2.03	< 0.01
Data presented as Mean ± Standard error. NUA: Normal umbilical artery, EDF: End-diastolic flow, REDF: Reverse end-diastolic flow, *Analysis of variance (ANOVA)				

**Table 3:** Results of comparing the first-hr mean glucose levels of newborns based on the results of umbilical artery Doppler ultrasonography


**UA Doppler ultrasonography**		**Mean difference ± standard error**	p-value*
**Normal UA Doppler**			
	**Absent EDF in UA Doppler**	15.21 ± 6.47	0.07
	**Reverse EDF in UA Doppler**	35.76 ± 9.96	< 0.01
**Absent EDF in UA Doppler**			
	**Normal UA Doppler**	-15.21 ± 6.47	0.07
	**Reverse EDF in UA Doppler**	20.54 ± 10.85	0.17
**Reverse EDF in UA Doppler**			
	**Normal UA Doppler**	-35.76 ± 9.96	< 0.01
	**Absent EDF in UA Doppler**	-20.54 ± 6.38	0.17
Data presented as Mean ± Standard error. EDF: End diastolic flow, UA: Umbilical artery, *Independent *t* test			

**Table 4:** Distribution of first-hr blood glucose based on the rate of AFI


**AFI**	**n**	**Minimum first-hr blood glucose**	**Maximum first-hr blood glucose**	**Mean ± SD**
** < 5 cm**	42	15	135	61.07 ± 4.85
**5-8 cm**	28	15	106	54.40 ± 3.99
** > 8 cm**	44	15	168	74.30 ± 5.18
P-value = 0.02, AFI: Amniotic fluid index, SD: Standard deviation				

**Table 5:** Results of comparing AFI based on the results of umbilical artery Doppler ultrasonography


**Umbilical artery Doppler ultrasonography**		**AFI**	p-value*
**Normal**			
	**Absent**	0.95 ± 0.85	0.54
	**Reverse**	4.28 ± 1.30	0.01
**Absent**			
	**Normal**	-0.95 ± 0.85	0.54
	**Reverse**	3.33 ± 1.40	0.07
**Reverse**			
	**Normal**	-4.28 ± 1.30	0.01
	**Absent**	-3.33 ± 1.40	0.07
Data shown as Mean difference ± Standard error. P-value = 0.05. AFI: Amniotic fluid index, *Independent *t* test			

**Table 6:** Frequency distribution of estimated weight percentile of the fetus depending on the cutoff point of 30 for first-hr neonatal blood glucose, mean of blood glucose, and Doppler ultrasonography


**Weight of fetus estimated by ultrasonography**		**EFW < 10%**	**EFW = 3-10%**	**EFW < 3%**	**Total**
**First-hr blood glucose < 30 (n [%])**		1 (3.3)	2 (3.5)	14 (5.2)	16 (14.0)
**First-hr blood glucose > 30 (n [%])**		29 (96.7)	18 (94.7)	51 (78.5)	98 (86.0)
**Minimum first-hr blood glucose**		30	16	15	-
**Maximum first-hr blood glucose**		135	110	168	-
**Mean of blood glucose ± SE**		73.00 ± 5.30	69.00 ± 6.04	59.33 ± 4.07	-
**Doppler ultrasonography (n)**					
	**Normal**	23	12	40	75
	**Absent**	4	6	19	29
	**Reverse**	3	1	6	10
EFW: Estimated fetus weight					

## 4. Discussion

UA Doppler ultrasonography shows normal or abnormal blood flow in the UA, which can be due to changes in the resistance of the placenta. Abnormal fetal hemodynamics, which can indicate a higher risk for abnormal pregnancy outcomes, can be detected using Doppler UA as a non-invasive method (17). In the present study, the relationship between UA Doppler ultrasonography and hypoglycemia in neonates with IUGR was investigated. According to the results, reverse EDF in UA Doppler is associated with a higher probability of hypoglycemia in the first hr after birth. In this study, hypoglycemia was found to be higher in neonates with borderline AFI compared to in neonates with normal AFI. Besides, reverse EDF in UA Doppler was associated with an increased probability of a reduction in amniotic fluid. However, there was no relationship found between UA Doppler ultrasonography and EFW in this study.

In a study conducted on 54 neonates with IUGR, neonates with abnormal Doppler were found to be at a higher risk of prenatal mortality and hospitalization in the intensive care unit (18). In another study conducted on 127 neonates with IUGR, prenatal mortality and other neonatal complications were higher in the abnormal UA Doppler group (19). In the present study, if we consider hypoglycemia as one of the IUGR-induced neonatal complications, in the reverse EDF in UA Doppler group, the probability of hypoglycemia was significantly higher than that of the normal Doppler ultrasonography group (p < 0.01) which is consistent with one of the previously mentioned studies (18). In the present study, the probability of hypoglycemia was found to be 21.9%, 5.3% and 3.3% in the EFW < 3%, EFW = 3-10%, and EFW < 10% groups, respectively. These data are in line with a retro-prospective study in which hypoglycemia was observed in 2.4% of the neonates weighing less than the 9 ${}^{\text{th}}$ percentile and in 4.5% and 19.1% of neonates weighing less than the 5 ${}^{\text{th}}$ percentile and 3 ${}^{\text{rd}}$ percentile, respectively (20).

In one study, neonatal complications in 372 healthy neonates were compared with those in 372 neonates with IUGR. It was found that the probability of hypoglycemia in neonates with IUGR was 5%, while it was 1% in the normal-growth neonates (21). In the present study, the probability of hypoglycemia in the neonates with IUGR was found to be 14%; however, healthy neonates were not studied due to ethical considerations.

In another study, the role of UA Doppler ultrasonography in predicting neonatal complications was studied in 134 neonates with IUGR. The prevalence of hypoglycemia in the two groups of absent and reverse UA Doppler ultrasonography was compared with that of the normal group. The probability of hypoglycemia was 9.1% in the absent and reverse Doppler ultrasonography group, while no hypoglycemia was observed in the normal Doppler ultrasonography group. The probability of oligohydramnios was 17.8% in the normal Doppler group and it was 64.3% in the abnormal Doppler group, and this difference was statistically significant (22). In our study, the probability of hypoglycemia was 8.0% in the normal group, 24.1% in the absent group, and 30.0% in the reverse Doppler group, and this difference was statistically significant (p = 0.03). The present study revealed that the probability of oligohydramnios was significantly higher in the reverse Doppler group than in the normal Doppler group (p = 0.01).

In another study on neonates with IUGR, it was shown that hypoglycemia was associated with weight at birth time, but it was not associated with UA Doppler ultrasonography results (23). These results are not in line with the present study in which the probability of hypoglycemia was found to be 3.3%, 5.3%, and 21.9% in the EFW < 10%, EFW = 3-10% and EFW < 3% groups, respectively. This difference was not statistically significant (p = 0.11). Also, no significant difference was found between the three Doppler groups in terms of estimated weight (p = 0.54). This result is not consistent with research conducted by McCowan and colleagues (18). In addition, in the present study, reverse Doppler was significantly associated with an increased probability of hypoglycemia, which is not in line with the results of previous research (23).

## 5. Conclusion 

Based on the results of this study, it can be concluded that postnatal hypoglycemia in neonates with IUGR is associated with UA Doppler ultrasonography during pregnancy. Reverse EDF in UA Doppler ultrasonography is significantly associated with an increased probability of hypoglycemia in the first hr of birth. It is recommended that blood glucose be closely monitored after birth in neonates with IUGR, especially if the UA Doppler is reverse. In this situation, the risk of hypoglycemia is higher and so the need for assessment of neonatal glucose is more important. Due to the numerous complications of IUGR with impaired Doppler ultrasound, it is recommended that other related complications, including the need for a cesarean section or labor induction, prenatal death, and hospitalization in the intensive care unit be investigated in future studies.

##  Conflict of Interest

The authors declare that there is no conflict of interest.
